# Rapid In Situ Near-Infrared Assessment of Tetrahydrocannabinolic Acid in Cannabis Inflorescences before Harvest Using Machine Learning

**DOI:** 10.3390/s24165081

**Published:** 2024-08-06

**Authors:** Jonathan Tran, Simone Vassiliadis, Aaron C. Elkins, Noel O. O. Cogan, Simone J. Rochfort

**Affiliations:** 1 School of Applied Systems Biology, La Trobe University, Bundoora, VIC 3083, Australia; noel.cogan@agriculture.vic.gov.au (N.O.O.C.); simone.rochfort@agriculture.vic.gov.au (S.J.R.); 2 Agriculture Victoria Research, AgriBio Centre, AgriBio, Melbourne, VIC 3083, Australia; simone.vassiliadis@agriculture.vic.gov.au (S.V.); aaron.elkins@agriculture.vic.gov.au (A.C.E.)

**Keywords:** prediction models, cannabinoids, regression, classification, portability, THCA, PLS, SVM, XGB

## Abstract

Cannabis is cultivated for therapeutic and recreational purposes where delta-9 tetrahydrocannabinol (THC) is a main target for its therapeutic effects. As the global cannabis industry and research into cannabinoids expands, more efficient and cost-effective analysis methods for determining cannabinoid concentrations will be beneficial to increase efficiencies and maximize productivity. The utilization of machine learning tools to develop near-infrared (NIR) spectroscopy-based prediction models, which have been validated from accurate and sensitive chemical analysis, such as gas chromatography (GC) or liquid chromatography mass spectroscopy (LCMS), is essential. Previous research on cannabinoid prediction models targeted decarboxylated cannabinoids, such as THC, rather than the naturally occurring precursor, tetrahydrocannabinolic acid (THCA), and utilize finely ground cannabis inflorescence. The current study focuses on building prediction models for THCA concentrations in whole cannabis inflorescences prior to harvest, by employing non-destructive screening techniques so cultivators may rapidly characterize high-performing cultivars for chemotype in real time, thus facilitating targeted optimization of crossbreeding efforts. Using NIR spectroscopy and LCMS to create prediction models we can differentiate between high-THCA and even ratio classes with 100% prediction accuracy. We have also developed prediction models for THCA concentration with a *R*^2^ = 0.78 with a prediction error average of 13%. This study demonstrates the viability of a portable handheld NIR device to predict THCA concentrations on whole cannabis samples before harvest, allowing the evaluation of cannabinoid profiles to be made earlier, therefore increasing high-throughput and rapid capabilities.

## 1. Introduction

*Cannabis sativa* L. is known for its medicinal and recreational uses, despite being used for thousands of years, its prohibition in many countries has hindered scientific research [1,2]. However, in recent years, the legalization of cannabis in some states and countries has led to a surge in studies exploring the potential benefits and risks of its use [3]. Advancements in cannabis research have prompted larger cultivation trials, aimed at exploring diverse phenotypic and chemovar variations, with the primary goal being to enhance production efficiencies and develop high-yielding cannabinoid cultivars [4]. Simultaneously, efforts are being made to simplify manual processing during cultivation and harvesting [4].

The two main cannabinoids of interest are cannabidiol (CBD) and delta-9-tetrahydrocannabinol (THC). CBD is used for the treatment of epilepsy, particularly in children [5], and as an analgesic and anti-anxiolytic [6]. THC has been investigated as a treatment for multiple sclerosis, spasticity, chronic pain, and posttraumatic stress disorder (PTSD) bringing THC to the forefront of medicinal cannabis’s focus [7,8,9]. THC and CBD are converted from tetrahydrocannabinolic acid (THCA) and cannabidiolic acid (CBDA), respectively, through a process called decarboxylation, which is typically achieved by heating cannabis inflorescences. These precursor compounds are naturally formed in the cannabis plants and found in abundance, where THC and CBD are present in relatively minor amounts, so it is important to target these THCA and CBDA compounds *in planta*, over their THC and CBD counterparts [10].

Traditional methods of cannabis analysis are performed using gas chromatography (GC) and liquid chromatography (LC), coupled with a mass spectroscopy (MS) as a detector [11], other methods use flame-ionization detection (FID) coupled with GC or an ultra-violet diode array detector coupled with LC [11,12]. Mass spectroscopy provides highly accurate and selective results; however, all forms and variations of analysis are expensive and slow compared to other techniques such as near-infrared (NIR) spectroscopy. NIR spectroscopy is a rapid and non-destructive technique capable of detecting molecular vibrations through organic bonds and discern them, however, it cannot fully elucidate chemical compounds and is non-selective for individual cannabinoids [13,14,15,16,17]. These spectra can be used to make cannabinoid content predictions or distinguish between cultivars once paired with high-quality quantitation data and multivariate statistical software [16,18].

Methods for using near-infrared spectroscopy for the analysis of cannabinoids in finely ground inflorescence material is well-researched and when combined with high-quality MS data can produce strong prediction models to predict cannabinoid content [13,14,15,16,17,19,20]. Sample preparation requirements can be reduced by leaving inflorescences whole or by coarsely grinding the tissues, as opposed to finely grinding the tissues which is necessary for LCMS and GCMS, thereby accelerating the sampling processes where high-throughput screening is needed. However, this is typically associated with loss in prediction model accuracy, as samples are less homogenous. This was illustrated by Deidda et al. (2021), where THC prediction models generated from finely ground inflorescences (*R*^2^ 0.93) outperformed coarsely ground material (*R*^2^ 0.74) [19]. Prior work compared coarsely ground inflorescences and finely ground inflorescences using a FT-NIR Bruker MPA II, where prediction models for 14 cannabinoids were produced. Partial least squares regression (PLS-R) models for CBDA, cannabinol (CBN), cannabinolic acid (CBNA) and cannabichromenic acid (CBCA) using finely ground inflorescences (*R*^2^ = 0.99, 0.71, 0.79 and 0.60, respectively) were comparable to those obtained using coarsely ground inflorescences (*R*^2^ = 0.98, 0.69, 0.79 and 0.59, respectively), while all other cannabinoid prediction models performed poorly (lower *R*^2^ values) when coarsely ground inflorescences were used to make predictions [16].

Multiple machine learning modelling techniques should be employed when developing prediction methods, as certain techniques may produce a more robust outcome than others. PLS models are a set of versatile statistical methods which can be used for regression (PLS-R) and classification (partial least squares discriminant analysis—PLS-DA). PLS methods require a data training set and accurate phenotypes (in this case, NIR spectra and quantitative LCMS data). The methods prioritize data variance that is correlated with the trait of interest. PLS has been used in a wide variety of areas such as genomics, chemometrics, environment studies and neuroinformatics [21]. Support vector machine (SVM) is a supervised machine learning algorithm works by maximizing the difference between finding the optimal hyperplane that best separates the data using support vectors. SVM has been used in cheminformatics, drug discovery and many other disciplines [22,23]. Extreme gradient boosting (XGB) is an ensemble machine learning algorithm. Ensemble learning is where the model is built upon multiple ‘weak’ learning models to create a more robust and accurate model which is better suited to solve problems than single models. XGB has been used in diagnostic classification of cancers, optimization of manufacturing and credit risk assessment models for financial institutions [24,25,26].

This research aims to provide prediction models for THCA in intact inflorescences of *C. sativa* grown under glasshouse conditions. NIR data were acquired using a portable handheld device (VIAVI MicroNIR) on whole, intact inflorescences to allow in situ, in planta scanning. Chemometrics included PLS-R, support vector machine regression (SVM-R) and extreme gradient boosting regression (XGB-R). This represents the first research to use a portable, handheld NIR device to distinguish between high THCA and even ratio chemovars in a glasshouse environment. High THCA was defined as classes that had a concentration ratio of CBDA to THCA lower than 1:1; and even ratio had a concentration ratio approximating 1:1 (CBDA:THCA ratio). This growing trial included 264 cannabis plants which is the largest sample size used for cannabinoid prediction models on whole cannabis inflorescences. This trial also has 88 unique genotypes to ensure a diverse and variable cannabinoid profile amongst the entire dataset to reduce population bias. The benefit of predicting cannabinoids using whole inflorescence is the elimination of needing to process the inflorescence into a finely ground powder, thus reducing cost and time, and increasing high-throughput capability.

## 2. Materials and Methods

### 2.1. Growth & Conditions

Cannabis plants were legally grown at a secured undisclosed location within a glasshouse by Cann Group (Cann Group Limited, Australia) under permit MC029-S01-C02 from the Office of Drug Control, Department of Health and Aged Care Australia. Plants grown during the vegetative state were exposed to a day-and-night cycle of 16:8 h. Plants during the flowering stages were exposed to a day-and-night cycle of 12:12 h. The temperature was maintained at 24–30 °C with 50–80% relative humidity. There were 88 unique genotypes cloned in triplicate to produce 264 cannabis plants. The chemovars present within population were high-producing THCA (n = 231) cultivars and even-ratio (n = 33) cultivars. Data collection took place over two days just prior to harvest. The data was collected at 10 am to 11 am each day at a temperature 25 °C with 60% relative humidity.

### 2.2. Instrumentation & Parameters

A VIAVI MicroNIR Onsite-W (VIAVI Solutions Inc., Scottsdale, AZ, USA) was utilized for scanning and data were collected within the 950–1650 nm (10,526–6060 cm^−1^) range, with a resolution of 6.2 nm. The handheld portable MicroNIR uses two integrated vacuum tungsten lamps with a 128-pixel InGaAs photodiode array detector. The dispersing element is a VIAVI linear variable filter (LVF) (VIAVI Solutions Inc., Scottsdale, AZ, USA) and the windowed measurement collar uses an antireflective sapphire window. The signal-to-noise ratio is 25,000 and integration time of 10 ms. The portability and field deploy-ability allows it to be used in the field or within growing facilities. The detector was thoroughly cleaned using 80% methanol (Thermo Fisher Scientific Inc., Waltham, MA, USA) (methanol:water) and a KimTech KimWipe tissue (Kimberly-Clark, Irving, TX, USA) between all scans until there was no residue left. All scans were performed on cannabis inflorescences before harvest, on the top apical inflorescence of each cannabis plant, in triplicate, scanning a different section of the top apical inflorescence each time and the resulting data averaged. The device was configured in diffuse reflectance mode with an integration time of 12 milliseconds and 100 scan counts. Data acquisition was performed using MicroNIR Pro v3.0 software (VIAVI Solutions Inc., Scottsdale, AZ, USA). In total, 264 cannabis samples were scanned (88 genotypes × 3 replicates per genotype).

### 2.3. Sample Preparation for LCMS Analysis

The top apical inflorescence of each cannabis plant was taken, dried for 72 h, and weighed to assess moisture loss. Each sample was then vacuum sealed and sent to Agriculture Victoria Research (Bundoora, Melbourne, Victoria, Australia) for LCMS analysis and stored at room temperature prior to sample preparation and analysis. All samples were freeze-dried for 48 h using a VirTis General Purpose Freeze Dryer (Scientific Products, Warminster, PA, USA), transferred into 15 mL grinding jars, placed in liquid nitrogen for 1 min and ground to fine powder using a SPEX SamplePrep 2010 Geno/Grinder (SPEX SamplePrep, Metuchen, NJ, USA) for 1 min at 1500 rpm. 10 mg of the fine powder was then subsampled and quantitatively analyzed by LCMS for cannabinoid content, method described by Elkins et al. (2019) [12].

### 2.4. Statistical Analysis

**Figure 1:**
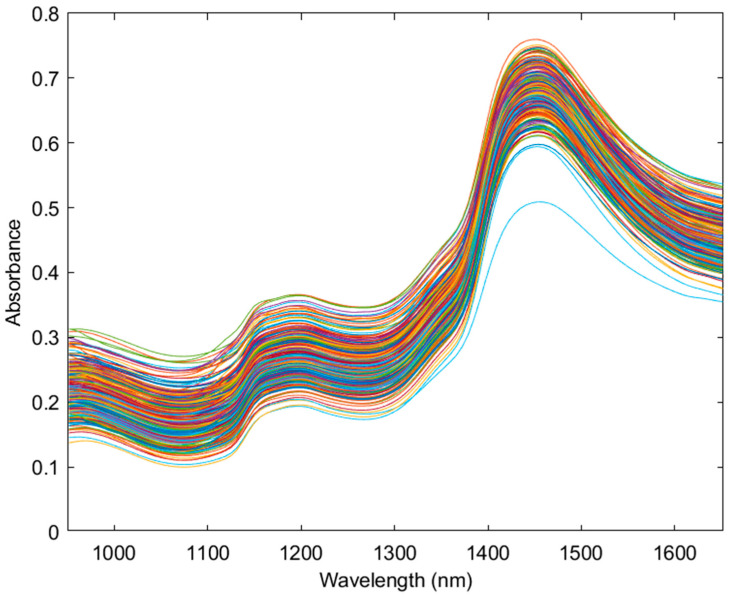
The raw near-infrared spectral data of the cannabis dataset (n = 264) from the 950 nm to 1650 nm range. Each color represents a unique scanned sample.

**Figure 2:**
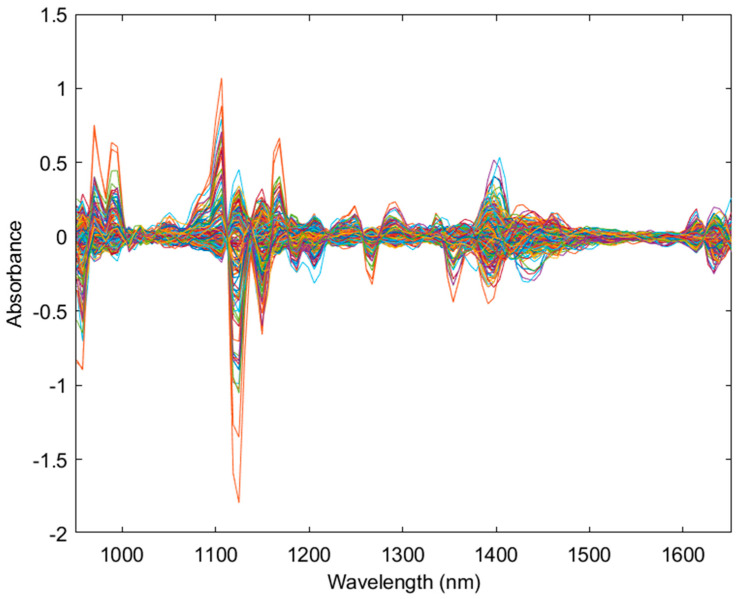
The pre-processed (detrend, standard normal variate, 2nd derivative and mean center) near-infrared spectral data of the cannabis dataset (n = 264) from the 950 nm to 1650 nm range. Each color represents a unique scanned sample.

VIAVI MicroNIR Onsite-W spectra data were exported from MicroNIR Pro v3.0 and imported into Excel (Microsoft Corporation, Redmond, WA, USA), where the data from the three scans were averaged. The averaged data was imported into MATLAB 2022a (Mathworks, Natick, MA, USA) with PLS-Toolbox 9.0 (Eigenvector Research Inc., Manson, WA, USA) for analysis. The full spectra range of the MicroNIR from 950–1650 nm (10,526–6060 cm^−1^) was utilized (Figure 1). For statistical modelling, pre-processing used a combination of detrend, 2nd order derivate, standard normal variate (SNV) and mean centering to obtain the strongest model based on *R*^2^ prediction value (Figure 2). Principal component analysis (PCA) was performed to check data quality and explore trends within the sample population. PLS-DA models were used to categorize chemovars as high THCA and even ratio, and PLS-R and SVM-R models were used to create predictive models for THCA. PLS, SVM and XGB models used LCMS quantitative data as dependent variables and NIR data from the VIAVI MicroNIR Onsite-W as independent variables. Venetian blinds cross-validation was used within 10 splits and blind thickness set to 1.

The dataset would be separated into calibration and validation sets where 75% (n = 198) of the data was used to train the model and 25% (n = 66) of the data was used to test the model. The Kennard-Stone algorithm was used to ensure the data selected for the calibration set was uniform and representative of the whole data set while the validation set contains samples that are interior and exterior to the calibration set. The algorithm achieves this by randomly seeking two sample with the data set with the largest distance measure using either Euclidean or Mahalanobis distance [27]. This eliminates extrapolation of the calibration model when applied to the validation test. The same calibration and validation dataset split was used for all models. To test for statistical significance, permutation tests (n = 200) were performed for all models (Wilcoxon, Sign Test and Rand *t*-test).

## 3. Results

### 3.1. Principal Component Analysis of NIR Data

**Figure 3:**
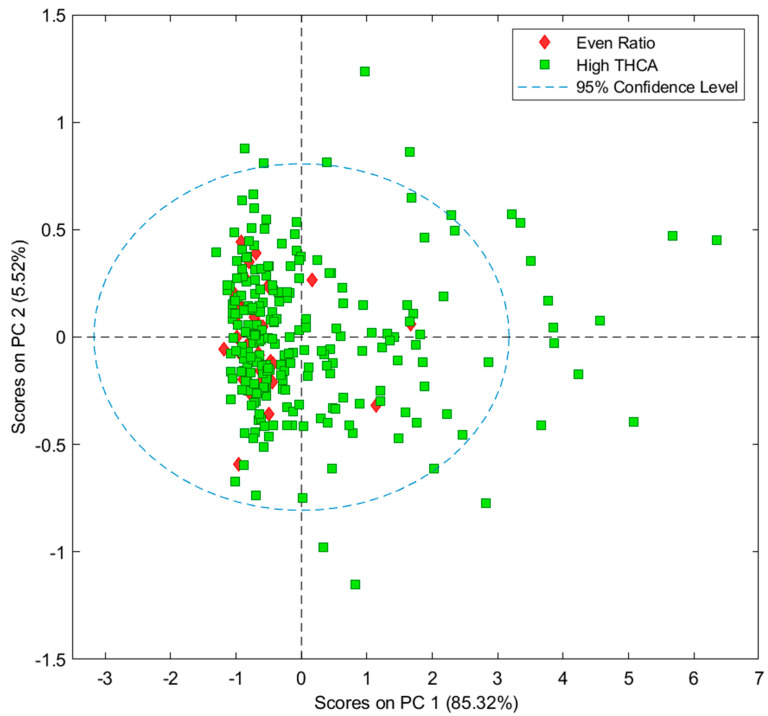
Scores of two principal components of the entire cannabis sample set (averaged data, n = 264). High THCA = green squares (n = 231), even ratio = red diamonds (n = 33).

PCA (Figure 3) was performed on the dataset to identify trends and assess data quality. The data is spread across principal component (PC) 1 and 2, with PC1 accounting for 85.72% of the variation within the dataset, but no clusters separating into even ratio and high THCA chemovars were identified.

Previously, Tran et al. [16] performed a PCA on their cannabis datasets and reported major clusters in previous cannabis datasets which are categorized into high THCA, high CBDA and even ratio chemovars. Clustering indicates that discriminant analysis and regression models will likely be exceptional for the related chemovar. In the current paper, the data does not cluster into chemovars (high THCA and even ratio). An explanation for the absence of clustering may be due to the lack of homogeneity of whole cannabis inflorescence compared to finely ground cannabis inflorescence, where the latter would have more exposed surface area and uniform cannabinoid distribution when performing scans on the MicroNIR.

### 3.2. Partial Least Squares Discriminant Analysis (PLS-DA) Modelling

**Figure 4:**
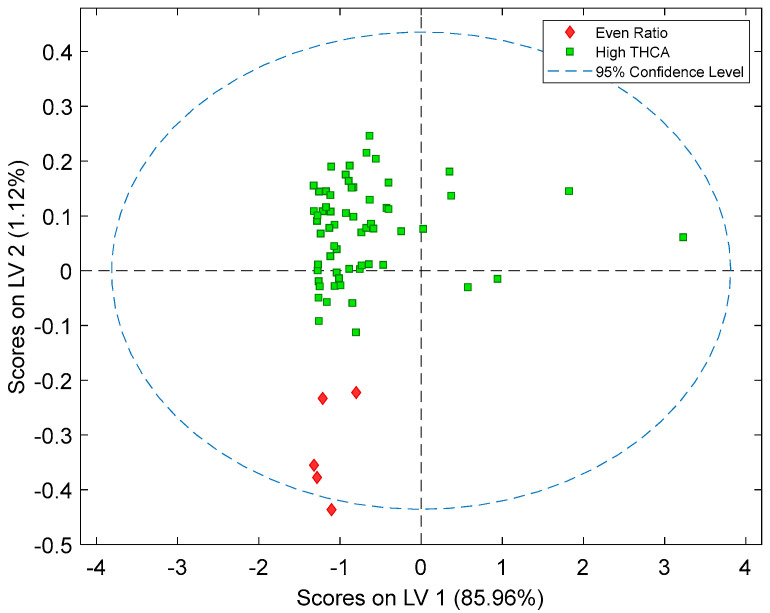
The PLS-DA score plot of two latent variables (LV 1 and LV 2) from the validation dataset (n = 66). Two clusters across LV2 were assigned as high-THCA = green squares (n = 61) and even ratio = red diamonds (n = 5).

**Figure 5:**
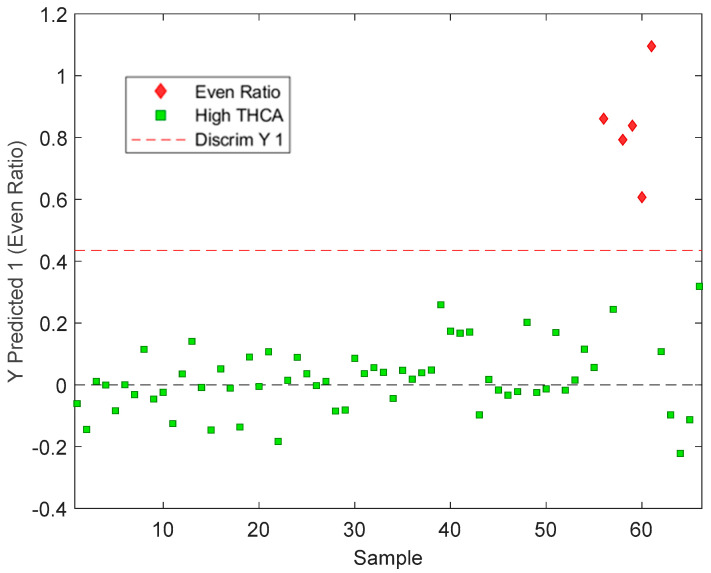
The PLS-DA classification of high-THCA = green squares (n = 61) and even ratio red diamonds (n = 5) classes of the cannabis validation dataset (n = 66).

PLS-DA models were able to predict cannabis samples as either high THCA or even-ratio classes with 100% accuracy (Class error Pred., 0%) (Table 1). High THCA was defined as a class that had a concentration ratio of CBDA to THCA lower than 1:1; and even ratio had a concentration ratio of more than 1:1 (CBDA:THCA ratio). The sample with highest CBDA:THCA ratio present was 1.723:1 (even ratio) and the lowest was 0.002:1 (high THCA), this data is available in Table S1. Sensitivity indicates how well the model can correctly classify samples as either high THCA or even ratio, whilst specificity measures how well the model can predict samples that are not high THCA or even ratio samples. The sensitivity and specificity values were equal to 1.00 for high-THCA and even ratio predictors, meaning a perfect discrimination between the two classes. The PLS-DA score plot of the validation dataset (Figure 4) showed clustering along the Latent Variable (LV) 2 with high THCA chemovars clustering together and the remaining even ratio chemovars distinctly separating towards the lower end of LV 2. This separation between the two chemovars is reflected in the classification predictions (Figure 5) where the validation results show that the PLS-DA model can discriminate between high THCA and even-ratio with 100% accuracy [28,29].

Birenboim et al. [18] performed classification predictions with NIR but utilized homogenized cannabis powder instead of whole cannabis inflorescence. The ability to be able to use NIR to scan directly onto whole cannabis inflorescence before harvest whilst obtaining 100% accuracy in predictions is of great added value to the cannabis industry as there will be less focus on processing of every cannabis inflorescence to evaluate their chemovar profile using more expensive processes such as LCMS or GCMS.

**Figure 6:**
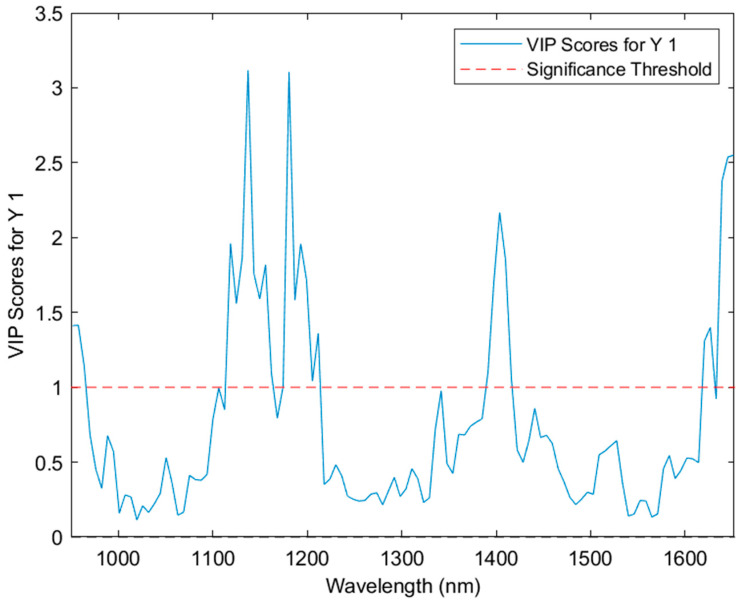
The VIP scores of the PLS-DA model across the near-infrared spectrum from 950 to 1650 nm.

The variable importance in projection (VIP) scores plot (Figure 6) displays the significance of each variable where scores of greater than one can be considered important. The VIP scores can be explained with respect to various organic functional groups; 950 nm (Ar-OH), 1150 nm (CH_2_ & CH_3_ groups), 1200 nm (CH groups), 1400 nm (CH and R-OH groups) and 1650 nm (Ar-CH bonds). The two compounds that factor heavily into these predictions are THCA and CBDA, which have Ar-OH, Ar-CH, CH and R-OH groups all which correspond to wavelengths on the NIR spectrum. THCA lacks one Ar-OH group whereas CBDA has two.

### 3.3. Partial Least Squares Regression (PLS-R), Support Vector Machine Regression (SVM-R) and XGBoost Regression (XGB-R) Modelling

Different pre-processing parameters were applied to optimize the training models of each modelling algorithm for THCA concentrations (Table 2). XGB-R produced the highest *R*^2^ calibration values (0.93, 1.00 and 1.00), followed by SVM-R (0.68, 0.78, 0.68) and PLS-R (0.62, 0.57, 0.62), however, when optimizing validation models, PLS-R (0.78, 0.75, 0.73) and SVM-R (0.77, 0.76, 0.74) models performed slightly better than the XGB-R (0.74, 0.59, 0.74) models.

Despite the XGB-R models exceptional performance with high *R*^2^ calibration values, this was not reflected in validation tests, reducing *R*^2^ prediction values to 0.74, 0.59 and 0.74. *R*^2^ prediction values of 0.74 is suitable for screening, however, a *R*^2^ prediction value of 0.59 is significant reduction from the *R*^2^ calibration value of 1.00, suggesting an overfitting of the training model. The calibration step is very important in selecting the best performing modelling algorithm and preprocessing parameters, but it is vital that validation tests are performed against the training model to ensure the model is not overfitted to ensure suitability for real-world applications.

From this, a PLS-R modelling algorithm (Figure 6), with an *R*^2^ prediction value of 0.78 and RPD (residual prediction deviation) of 2.08 was selected as the best prediction model and was suitable for screening THCA concentrations in whole cannabis inflorescence [30] whilst having a low prediction bias of −0.68. It is to be noted that a positive value for prediction bias indicates an overfitting of a model, whilst a negative value for prediction bias indicates the underfitting of a model. Therefore, values nearing zero are ideal and indicate a lower bias. Ultimately, PLS-R produced the strongest model and the second lowest prediction bias.

THCA concentration across the cannabis population ranged from 37.29 to 229.87 mg/g as shown in Table S1. The residual prediction deviation (RPD) is typically measured as a goodness of fit of a prediction model, however as Minasny et al. (2013) [31] mentions, RPD is the same measure as *R*^2^, instead, a more suitable measure would be the ratio of standard error laboratory (SEL) versus the standard error prediction (SEP) [16,32]. The SEL and SEP ratio reported in supplementary files (Table S2) shows poor precision, but this is expected due to the heterogeneity of the cannabis samples analyzed via the NIR. The prediction error average was 13% for the validation set (Table S3) and defines the limits of this method, but it will still be a suitable method for general screening of whole cannabis inflorescence for THCA concentrations, especially for the cannabis industry where high THCA chemovars are the focus for both recreational and medicinal markets. Previous research which developed cannabinoid prediction models using dried finely ground cannabis inflorescence material have lower errors and stronger prediction models compared to whole inflorescence, likely due to a more homogenous sample with greater surface area compared to inflorescences in situ [13,14,15,16,17,19]. Another factor is the presence of water in the in-situ samples. As each scan was performed on a cannabis inflorescence before harvest, there was no drying step to remove moisture.

Water exhibits absorption bands in the NIR range at 1400–1450 nm. These bands correspond to hydroxyl groups (O-H) in water molecules which overlap with signals from hydrocarbon (C-H) and alcohol (R-OH) groups within the spectra and potentially cause inaccurate predictions. This provides challenges in creating robust prediction models, but the current study demonstrates that, in practice, this is a minor issue. Trimming the NIR spectra and removing the 1400–1450 nm range is alternative if eliminating the water interference is priority, however this resulted in significantly lower *R*^2^ values (Table S4, Figure S1), likely due to the fact the omitted range still contain valuable peak data that corresponds to organic bonds found in cannabinoid compounds. The purpose of scanning inflorescences which are in situ, pre-harvest, intact and non-dried is intentional, as these conditions emulate the practices performed by industry cultivators.

Deidda et al. [19] (2021) used a MicroNIR (950–1650 nm) where three prediction models for THC concentration were developed for whole cannabis (0.93), coarsely ground (0.76) and finely ground (0.77) inflorescences. In the same study a, NIR-S-G1 (900–1700 nm), also a handheld NIR device, was used but showed contradictory results where the *R*^2^ prediction values were reversed: whole cannabis (0.73), coarsely ground inflorescences (0.74) and finely ground (0.93). Typically, the more homogenous a sample is, the more surface area is exposed for the NIR detector to scan organic compounds giving stronger *R*^2^ prediction values. When a sample is heterogenous, a worse *R*^2^ prediction value would be expected as there would be more sample variance due to the lack of uniformity in the cannabinoids as they are distributed across a whole cannabis inflorescence. This was shown in previous work by Tran et al. [16] (2023) when comparing coarsely ground and finely ground inflorescences, where finely ground produced stronger PLS-R models using a VIAVI MicroNIR Onsite-W and FT-NIR Bruker MPA II. In addition, Deidda et al. employed genetic algorithm (GA) for variable selection which can be prone to overfitting. This occurs when a dataset contains over 200 variables (each wavelength number), which may overestimate a model’s performance and which may produce results not generalized enough to real world samples [33,34]. This current manuscript did not include GA results for reasons above, but it is worth noting that the *R*^2^ performance was comparable with that of the PLS-R results in Table 2. The dataset was reduced to 36 samples to replicate the sample size of Deidda’s study, but it resulted in poorer *R*^2^ values (0.59) and significant prediction bias (8.03) (Table S5, Figure S2).

**Figure 7:**
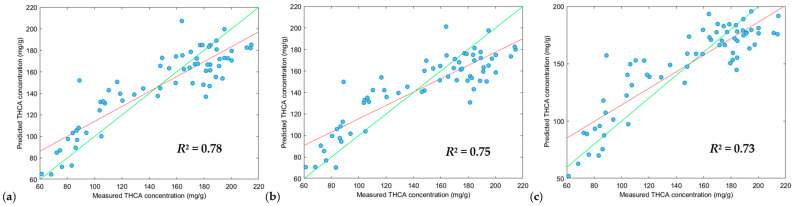
Plot of measured values versus the predicted values of THCA of the validation dataset (n = 66) using the PLS-R tool. Green line: line of best fit from reference data, red line: line of best fit from predicted data. Scatter correction and derivative settings: (**a**) DT, SNV and MC (2, 2, 5); (**b**) DT, SNV and MC (2, 2, 7); (**c**) DT, SNV and MC (2, 2, 3). Preprocessing parameters. DT: detrend; SNV: standard normal variate; MC: mean centering.

**Figure 8:**
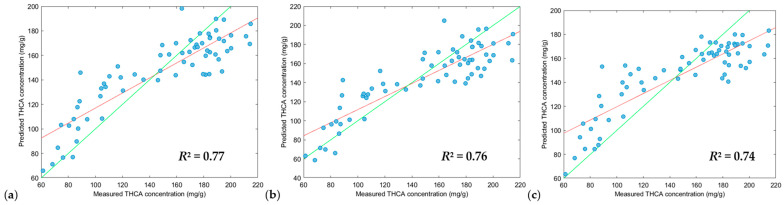
Plot of measured values versus the predicted values of THCA of the validation dataset (n = 66) using the SVM-R tool. Green line: line of best fit from reference data, red line: line of best fit from predicted data. Scatter correction and derivative settings: (**a**) DT, SNV and MC (2, 2, 5); (**b**) DT, SNV and MC (2, 2, 7); (**c**) DT, SNV and MC (2, 2, 3). Preprocessing parameters. DT: detrend; SNV: standard normal variate; MC: mean centering.

**Figure 9:**
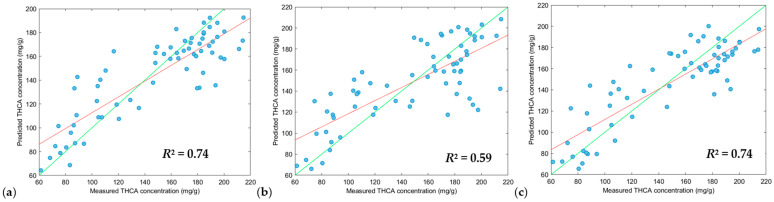
Plot of measured values versus the predicted values of THCA of the validation dataset (n = 66) using the XGB-R tool. Green line: line of best fit from reference data, red line: line of best fit from predicted data. Scatter correction and derivative settings: (**a**) DT, SNV and MC (2, 2, 5); (**b**) DT, SNV and MC (2, 2, 3); (**c**) DT and MC (2, 2, 5). Preprocessing parameters. DT: detrend; SNV: standard normal variate; MC: mean centering.

In previous literature, cannabinoid prediction models via NIR have shown limitations which calls for more robust models. This current manuscript improves on the model by containing 264 cannabis samples with 88 unique genotypes, making it the largest sample size used for predicting THCA concentrations from whole cannabis inflorescences. We have also conducted our analysis on in situ whole inflorescences before harvest, meaning the inflorescences are still attached to the plant with no sample handling processes. Finally, the current study performs no pre-treatment, where the cannabis inflorescences have not undergone any heating or decarboxylation step, leaving much of the THCA in its acid form rather than its neutral form, THC. This study furthers the cannabis industry by demonstrating that a handheld NIR system is suitable for the rapid determination of THCA concentration on in situ inflorescences, removing the need for extended drying periods and complex analyses. See Figure 7, Figure 8 and Figure 9.

## 4. Conclusions

The focus of this study was to expand upon the predictive capabilities using NIR spectroscopy to produce prediction models for THCA concentrations and predict between high THCA and even-ratio chemovars on whole cannabis inflorescence. The VIAVI MicroNIR Onsite-W as selected for its portability, rapid acquisition, and low cost. PLS-DA models were 100% accurate for defining cannabis samples as either high THCA or even ratio chemovar. PLS-R, SVM-R and XGB-R models were used to predict THCA concentrations, where PLS-R produced the best *R*^2^ prediction with low prediction bias. This method could be extended to predict other cannabinoids, provided there are sufficient sample numbers, and a range of cannabinoid concentrations present within the training set. The practicality of scanning whole cannabis inflorescences has improved the high-throughput capability by eliminating the need for grinding or processing the tissue down to fine powders resulting in reduced cost and time, remembering to consider that recalibrations should be performed based on temperature, humidity, and light changes. Scanning in real-time, allows for researchers, auditors, and cultivators to derive results onsite without needing to take samples to an analytical laboratory. This greatly benefits cultivators, as they can rapidly chemotype their plants and transfer their high-performing THCA plants into manufacturing glasshouses. In addition, real time, in situ screening may allow optimization of harvest times based on cannabinoid concentration, though this would require further research to demonstrate.

## Figures and Tables

**Table 1:** Calibration (n = 198) and validation (n = 66) values of partial least squares discriminant analysis (PLS-DA) models on the ratio of cannabinoid compounds analyzed by the VIAVI MicroNIR Onsite-W instrument.

	High THCA ^1^	Even Ratio ^2^
Sensitivity (Cal) ^3^	1.00	1.00
Specificity (Cal)	1.00	1.00
Sensitivity (CV) ^3^	1.00	1.00
Specificity (CV)	1.00	1.00
Sensitivity (Pred) ^3^	1.00	1.00
Specificity (Pred)	1.00	1.00
Class. Err ^4^ (Cal)	0.00	0.00
Class. Err (CV)	0.00	0.00
Class. Err (Pred)	0.00	0.00
RMSEC ^5^	0.15	0.15
RMSECV ^6^	0.15	0.15
RMSEP ^7^	0.12	0.12
Bias	0.00	0.00
CV Bias	0.00	0.00
Pred Bias	0.01	−0.01
*R*^2^_Cal_ ^8^	0.83	0.83
*R* ^2^_CV_	0.82	0.82
*R*^2^_Pred_ ^9^	0.79	0.79

^1^ THCA: tetrahydrocannabinolic acid; high THCA < 1:1. ^2^ Even ratio > 1:1 (1:1 = CBDA:THCA ratio). ^3^ Cal: calibration; CV: cross validation; Pred: prediction. ^4^ Class. Err.: classification error. ^5^ RMSEC: root-mean square error of calibration. ^6^ RMSECV: root-mean-square error of cross-validation. ^7^ RMSEP: root-mean-square error of prediction. ^8^ *R*^2^: coefficient of determination. ^9^ *R*^2^_Pred_: coefficient of regression of measured data vs. predicted data; Permutation testing (n = 200) using Wilcoxon, Sign test and Rand *t*-test test returned *p*-value < 0.05 for cross-validation (Figure S3a).

**Table 2:** The partial least squares regression (PLS-R), support vector machine regression (SVM-R) and extreme gradient boosting regression (XGB-R) models for the determination of THCA concentration.

Model	Figure Key	Region (nm) ^1^	Scatter Correction ^2^	Derivative ^2a^	N ^3^	RMSEC ^4^	*R*^2^_Cal_ ^5^	RMSECV ^6^	*R*^2^_CV_ ^7^	RMSEP ^8^	Pred Bias ^9^	*R*^2^_Pred_ ^10^	RPD ^11^
PLS-R	(a)	950–1650	DT, SNV and MC	2, 2, 5	264	26.34	0.62	28.87	0.54	21.49	−0.68	0.78	2.08
	(b)	950–1650	DT, SNV and MC	2, 2, 7	264	28.00	0.57	30.49	0.50	23.49	−2.61	0.75	1.91
	(c)	950–1650	DT, SNV and MC	2, 2, 3	264	26.41	0.62	30.93	0.48	23.34	0.77	0.73	1.93
SVM-R	(a)	950–1650	DT, SNV and MC	2, 2, 5	264	24.87	0.68	28.48	0.56	22.95	−1.47	0.77	1.96
	(b)	950–1650	DT, SNV and MC	2, 2, 7	264	23.87	0.70	29.40	0.53	22.49	−3.38	0.76	2.00
	(c)	950–1650	DT, SNV and MC	2, 2, 3	264	25.11	0.68	30.34	0.51	24.87	−1.80	0.74	1.81
XGB-R	(a)	950–1650	DT, SNV and MC	2, 2, 5	264	12.27	0.93	31.10	0.48	23.31	−3.46	0.74	1.88
	(b)	950–1650	DT, SNV and MC	2, 2, 3	264	0.02	1.00	34.61	0.37	28.77	0.44	0.59	1.56
	(c)	950–1650	DT and MC	2, 2, 5	264	0.25	1.00	34.09	0.38	23.02	−1.64	0.74	1.95

^1^ nm: wavelength in nanometers. ^2^ Preprocessing parameters. DT: detrend; SNV: standard normal variate; MC: mean centering. ^2a^ Derivative pre-treatment: the first digit is the polynomial order; the second digit is the derivative order, and the third digit is the data point gap which the derivative is calculated. ^3^ N: number of unique samples (Dataset was split using the Kennard-Stone algorithm into a calibration (n = 198) and validation set (n = 66) and used for all models. ^4^ RMSEC: root mean standard error of calibration. ^5^ *R*^2^_Cal_: coefficient of determination of calibration. ^6^ RMSECV: root mean standard error of cross validation. ^7^ *R*^2^_CV_: coefficient of determination of cross validation. ^8^ RMSEP: root mean standard error of prediction. ^9^ Pred Bias: calculated prediction bias. ^10^ *R*^2^_Pred_: coefficient of regression of measured data vs. predicted data; Permutation testing (n = 200) using Wilcoxon, Sign test and Rand *t*-test returned *p*-value < 0.05 for cross-validation results (Figure S3). ^11^ RPD: residual prediction deviation.

## Data Availability

Raw data are available upon request.

## Supplementary Materials

The following supporting information can be downloaded at: https://www.mdpi.com/article/10.3390/s24165081/s1, Table S1. The liquid chromatography mass spectra data of CBDA and THCA concentrations, ratios and chemovar classification within the cannabis dataset, Table S2. Standard error laboratory versus the standard error prediction for THCA, Table S3. Error of prediction values for the validation dataset used to test the PLS (*R*^2^ = 0.78) prediction model. Table S4. The partial least squares regression (PLS-R) models for the determination of THCA concentration where the water peak region (1400–1450 nm) from the NIR spectra has been removed. Table S5. The partial least squares regression (PLS-R) models for the determination of THCA concentration where the sample size (n = 36) has been reduced. Figure S1. Plot of measured values versus the predicted values of THCA of the validation dataset using the PLS-R tool where the water peak region (1400–1450 nm) from the NIR spectra has been removed. Figure S2. Plot of measured values versus the predicted values of THCA of the validation dataset using the PLS-R tool where sample size (n = 36) is reduced. Figure S3. The permutation results of each discriminant and regression model.
