# Supplementary material for: Rapid In Situ Near-Infrared Assessment of Tetrahydrocannabinolic Acid in Cannabis Inflorescences before Harvest Using Machine Learning
Source: Sensors (Basel). 2024 Aug 6;24(16):5081. doi: 10.3390/s24165081 (PMC11360504; doi:10.3390/s24165081)
Supplement: Supplementary file 1 [file sensors-24-05081-s001.zip › Figure S2.pdf]

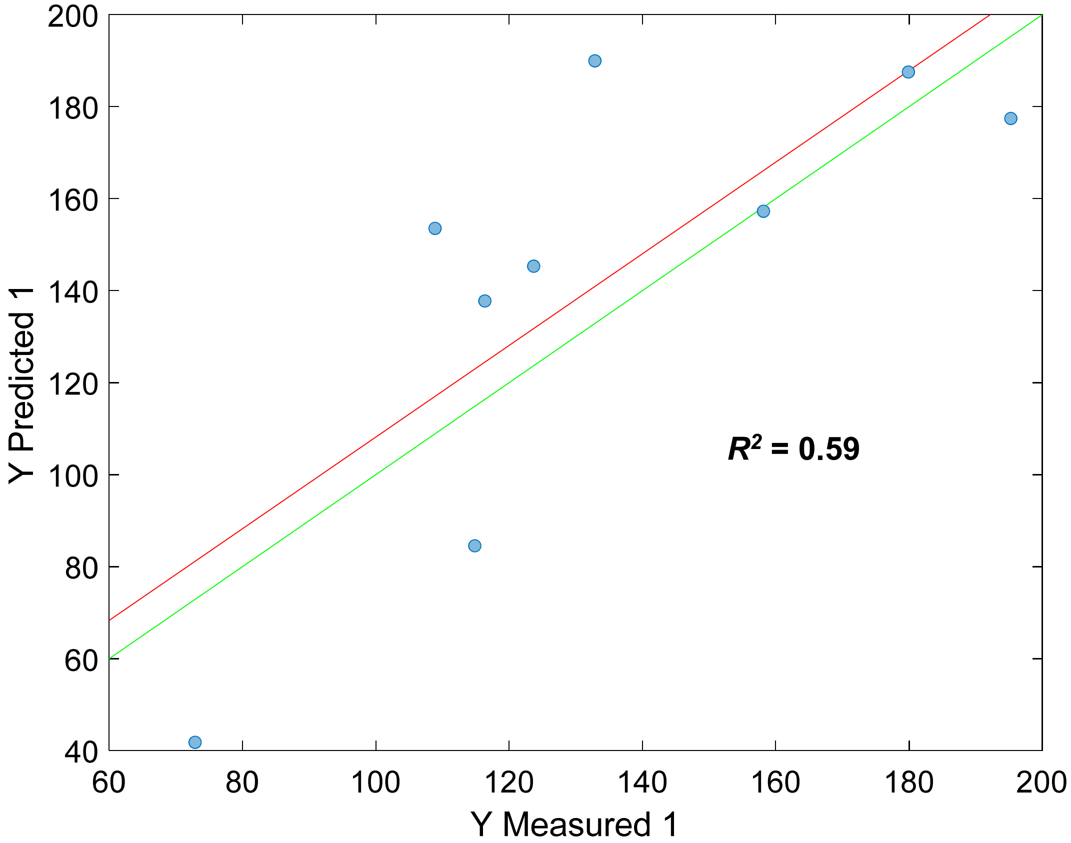

Figure S2. Plot of measured (n = 27) values versus the predicted (n = 9) values of THCA of the validation dataset using the PLS-R tool where sample size (n = 36) is reduced. Green line: line of best fit from reference data, red line: line of best fit from predicted data.
