# Supplementary material for: Rapid In Situ Near-Infrared Assessment of Tetrahydrocannabinolic Acid in Cannabis Inflorescences before Harvest Using Machine Learning
Source: Sensors (Basel). 2024 Aug 6;24(16):5081. doi: 10.3390/s24165081 (PMC11360504; doi:10.3390/s24165081)
Supplement: Supplementary file 1 [file sensors-24-05081-s001.zip › Figure S3.pdf]

Permutation Results

```
Probability of Model Insignificance vs. Permuted Samples
For model with 4 component(s)
________________________________
Y-column:  1
                  Wilcoxon     Sign Test     Rand t-test
Self-Prediction:   0.003         0.043          0.005
Cross-Validated:   0.001         0.016          0.005
________________________________
Y-column:  2
                  Wilcoxon     Sign Test     Rand t-test
Self-Prediction:   0.003         0.043          0.005
Cross-Validated:   0.001         0.016          0.005

Values less than 0.05 indicate the model is
significant at the 95% confidence level.
```

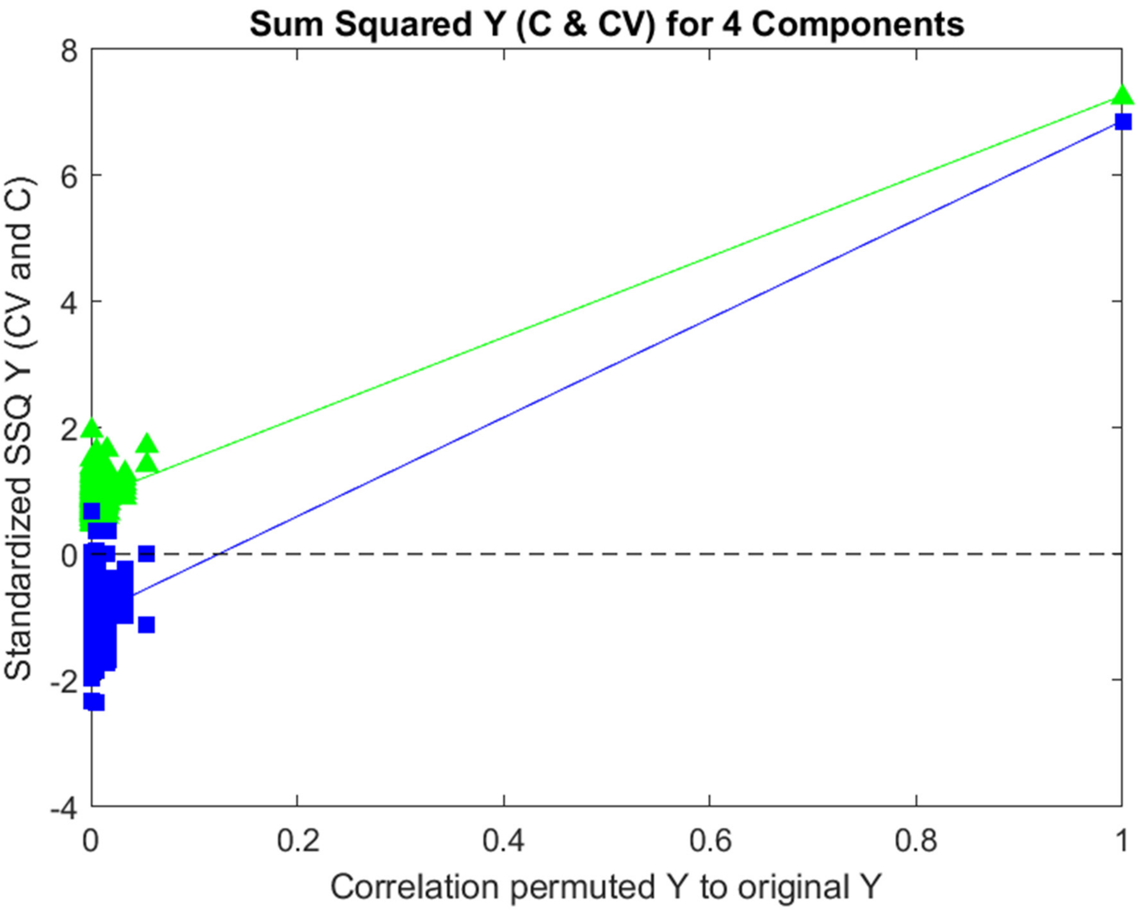

Figure S3a. Permutation test (n=200) of PLS-DA model Y-column 1 and 2.

Permutation Results

```
Probability of Model Insignificance vs. Permuted Samples
For model with 5 component(s)

_________________________________
Y-column:  1
                  Wilcoxon     Sign Test     Rand t-test
Self-Prediction:   0.000        0.000          0.005
Cross-Validated:   0.000        0.000          0.005

Values less than 0.05 indicate the model is
significant at the 95% confidence level.
```

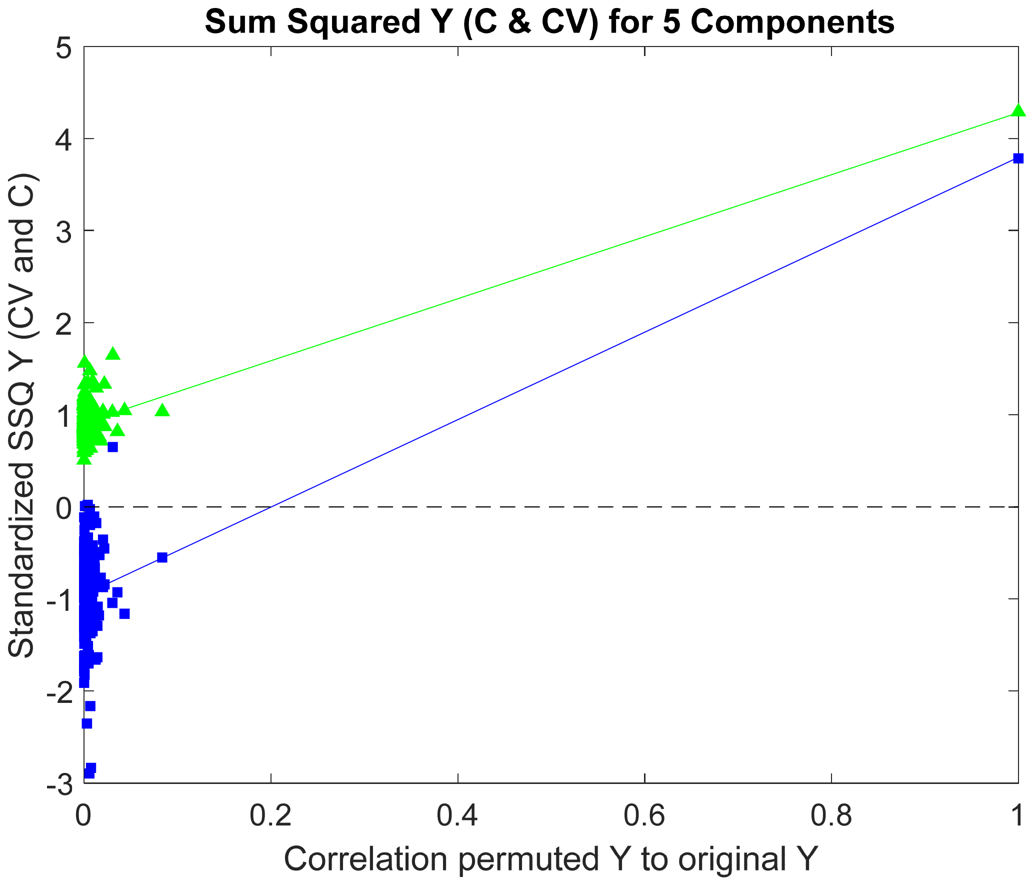

Figure S3b. Permutation test (n=200) of (a) PLS-R model where the R^2 prediction = 0.78

Permutation Results

```
Probability of Model Insignificance vs. Permuted Samples
For model with 5 component(s)
_________________________________
Y-column:  1
                     Wilcoxon     Sign Test     Rand t-test
Self-Prediction:      0.000         0.000          0.005
Cross-Validated:      0.000         0.000          0.005

Values less than 0.05 indicate the model is
significant at the 95% confidence level.
```

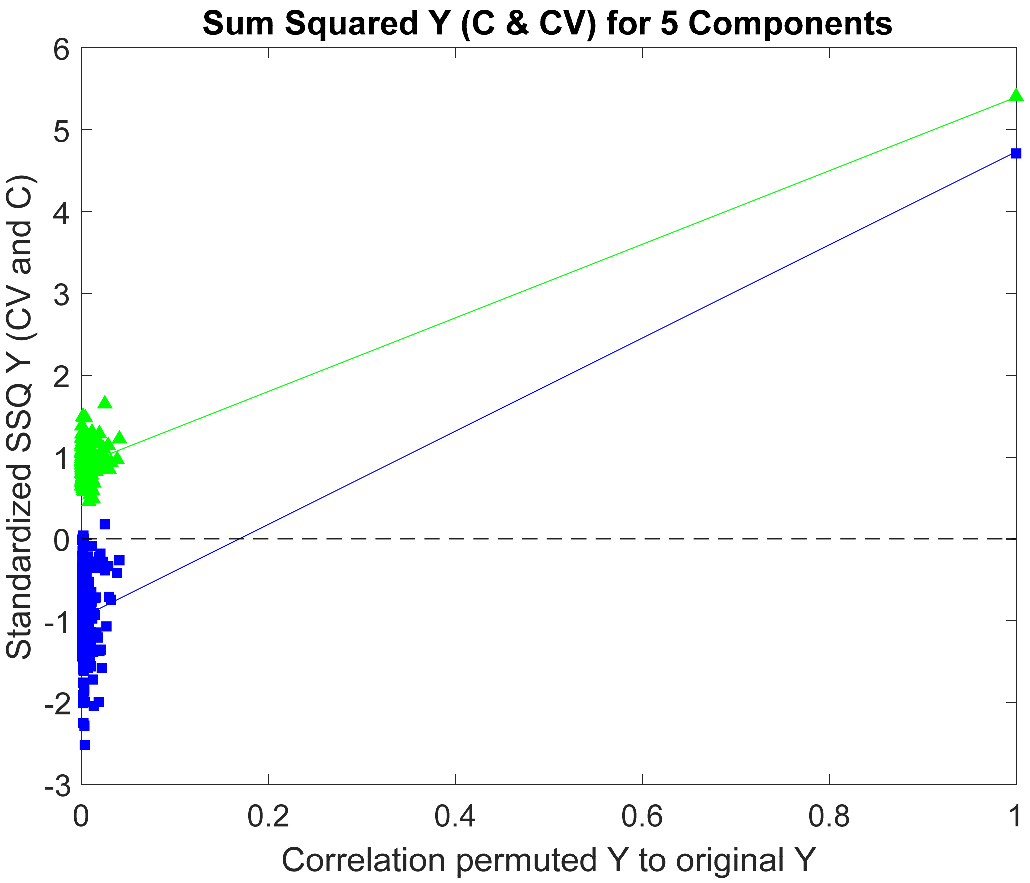

Figure S3c. Permutation test (n=200) of (b) PLS-R model where the R^2 prediction = 0.75

Permutation Results

```
Probability of Model Insignificance vs. Permuted Samples
For model with 4 component(s)
_________________________________
Y-column:  1
                  Wilcoxon     Sign Test     Rand t-test
Self-Prediction:   0.000        0.000          0.005
Cross-Validated:   0.000        0.000          0.005

Values less than 0.05 indicate the model is
significant at the 95% confidence level.
```

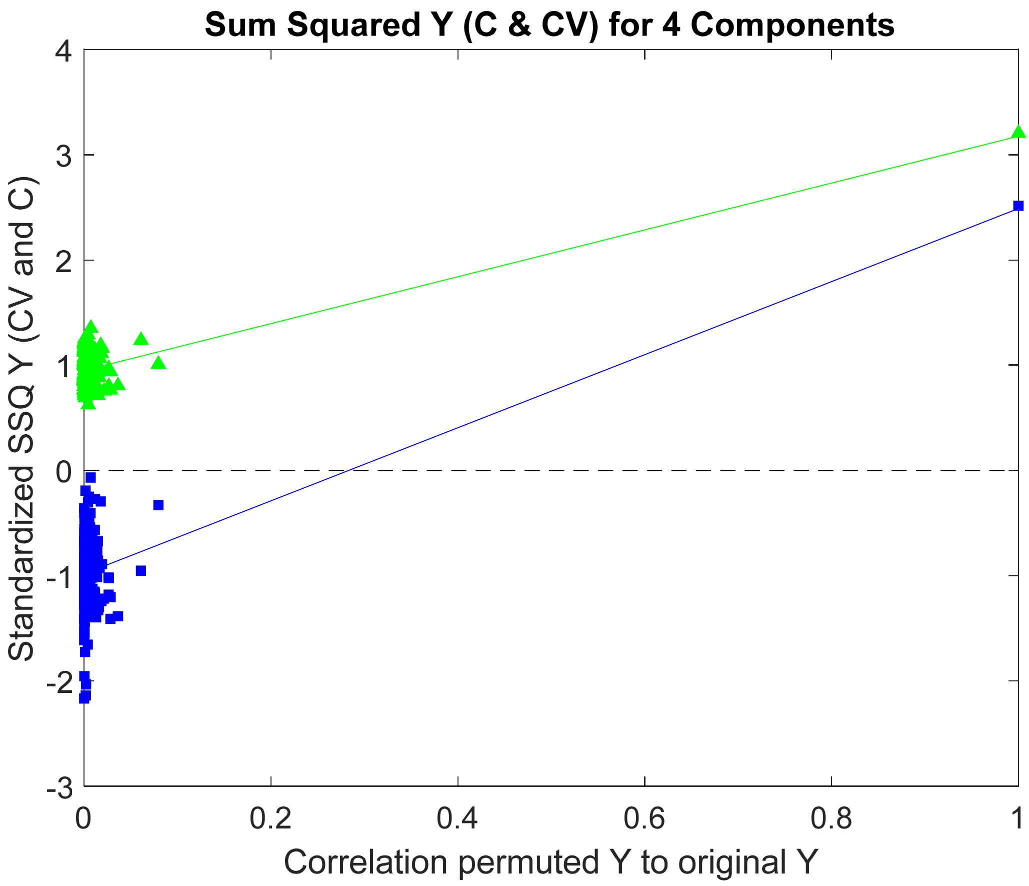

Figure S3d. Permutation test (n=200) of (c) PLS-R model where the R^2 prediction = 0.73

Permutation Results

```
Probability of Model Insignificance vs. Permuted Samples

_________________________________
Y-column:  1
                   Wilcoxon      Sign Test      Rand t-test
Self-Prediction:    0.111         0.117           0.009
Cross-Validated:    0.000         0.000           0.005

Values less than 0.05 indicate the model is
significant at the 95% confidence level.
```

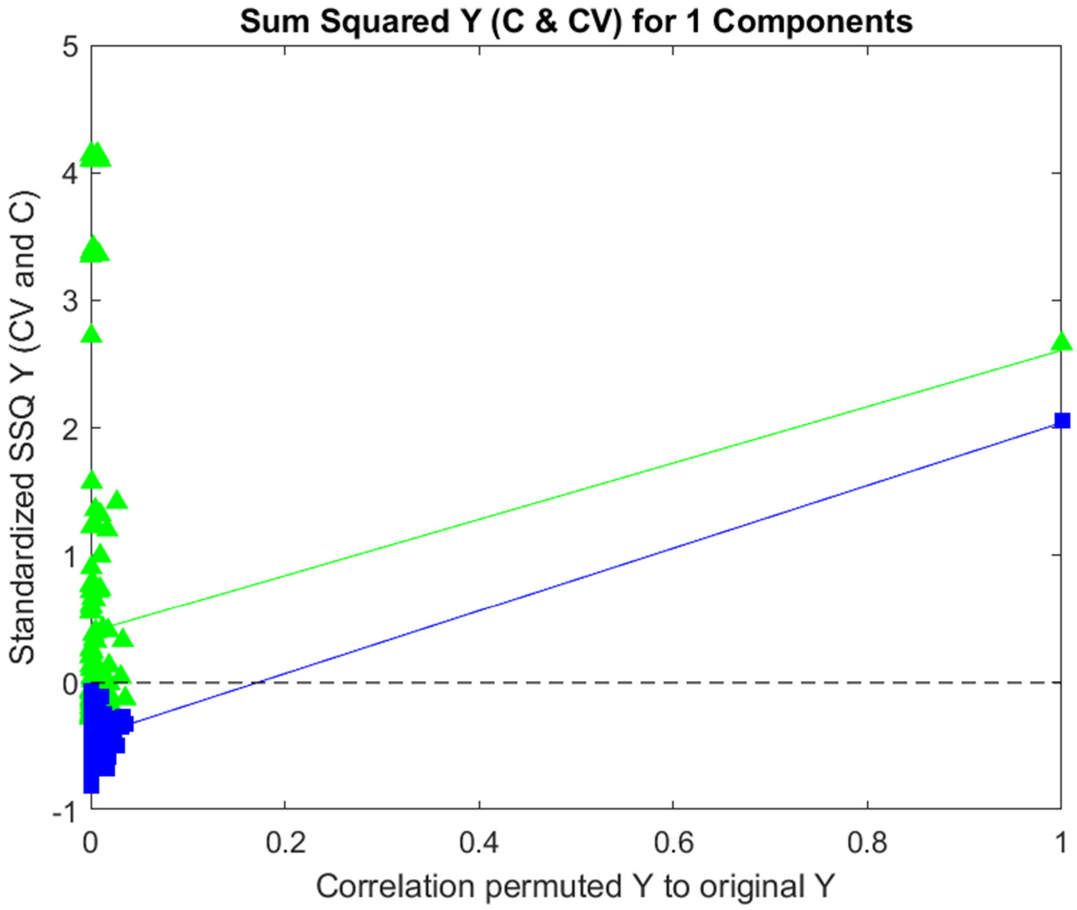

Figure S3e. Permutation test (n=200) of (a) SVM-R model where the R^2 prediction = 0.77

Permutation Results

```
Probability of Model Insignificance vs. Permuted Samples

__________________________________
Y-column:  1
                        Wilcoxon      Sign Test      Rand t-test
Self-Prediction:         0.035         0.036           0.005
Cross-Validated:         0.000         0.000           0.005

Values less than 0.05 indicate the model is
significant at the 95% confidence level.
```

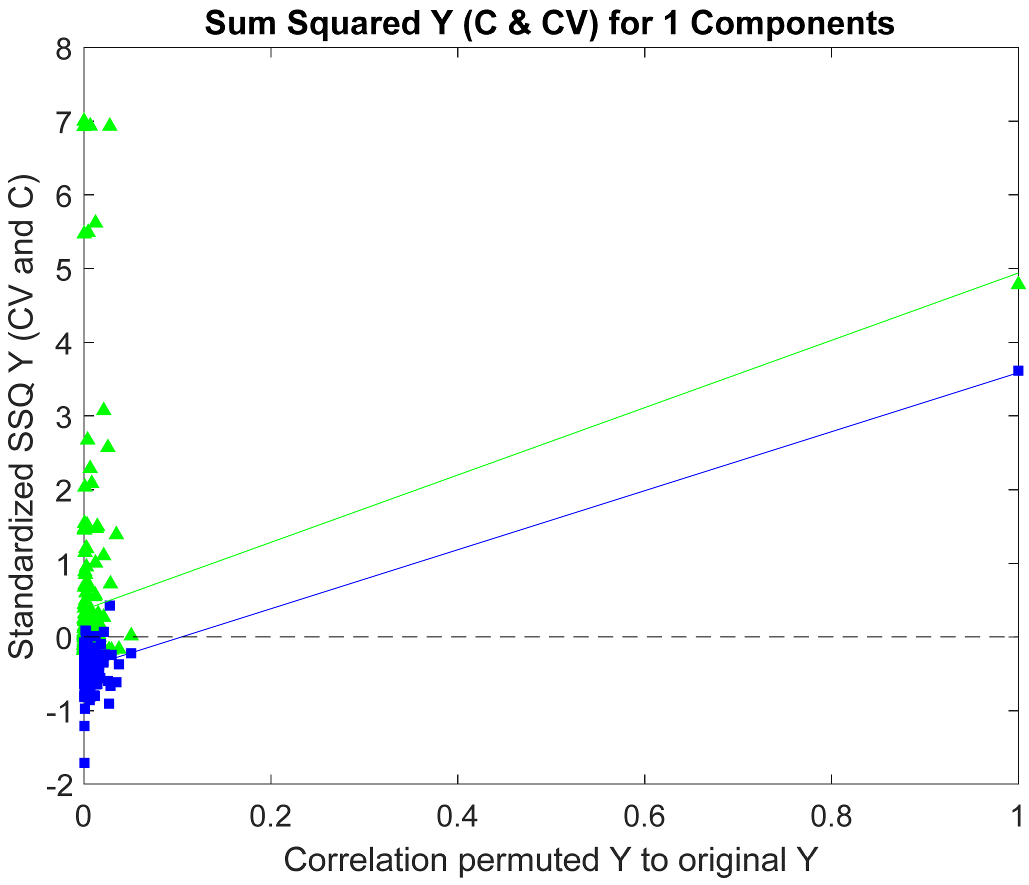

Figure S3f. Permutation test (n=200) of (c) SVM-R model where the R^2 prediction = 0.76

Permutation Results

Probability of Model Insignificance vs. Permuted Samples

Y-column: 1

	Wilcoxon	Sign Test	Rand t-test
Self-Prediction:	0.100	0.100	0.005
Cross-Validated:	0.000	0.000	0.005

Values less than 0.05 indicate the model is significant at the 95% confidence level.

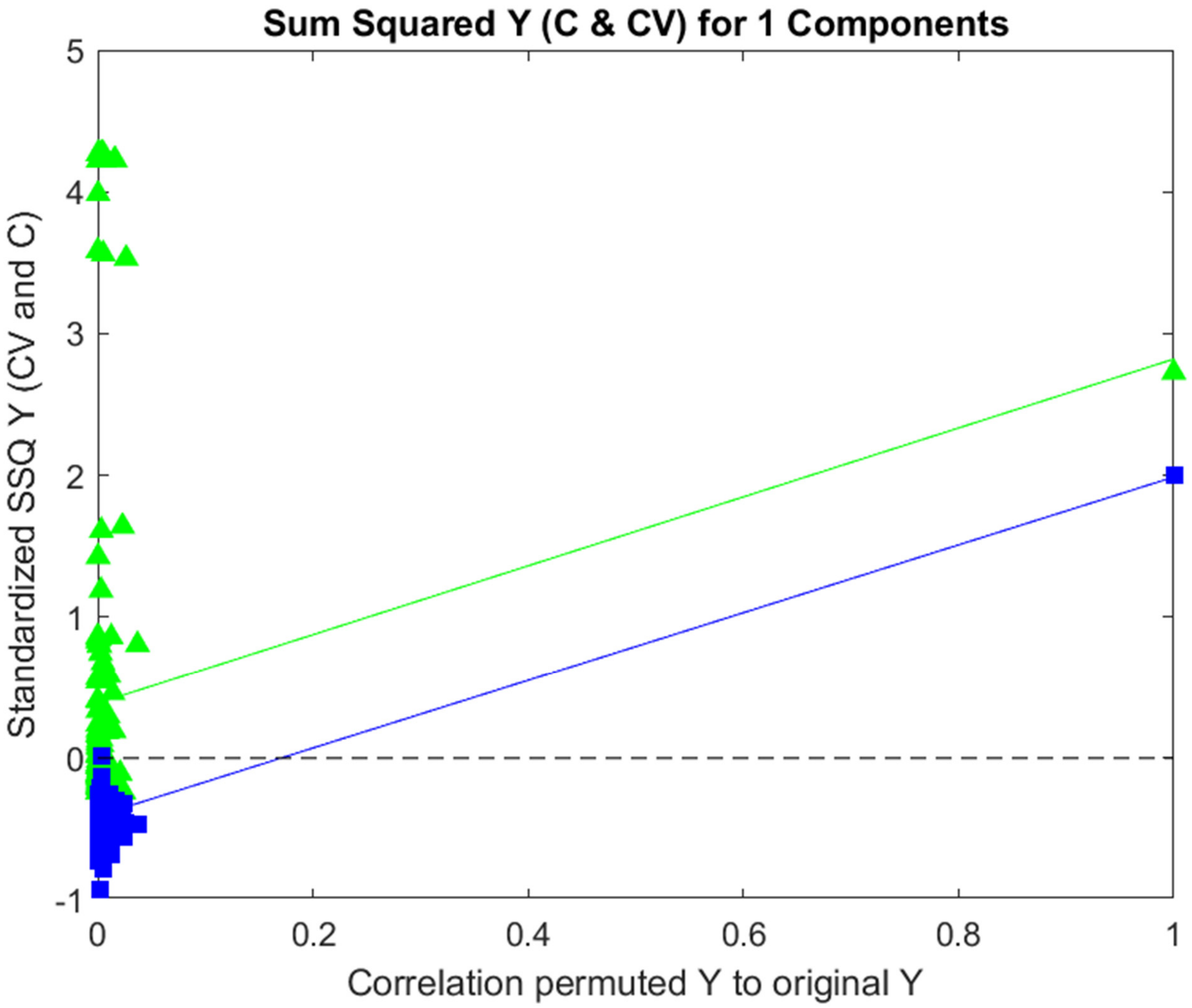

Figure S3g. Permutation test (n=200) of (b) SVM-R model where the R^2 prediction = 0.74

Permutation Results

```
Probability of Model Insignificance vs. Permuted Samples

Y-column:  1
                    Wilcoxon      Sign Test      Rand t-test
Self-Prediction:     0.000         0.000           0.005
Cross-Validated:     0.000         0.000           0.005

Values less than 0.05 indicate the model is
significant at the 95% confidence level.
```

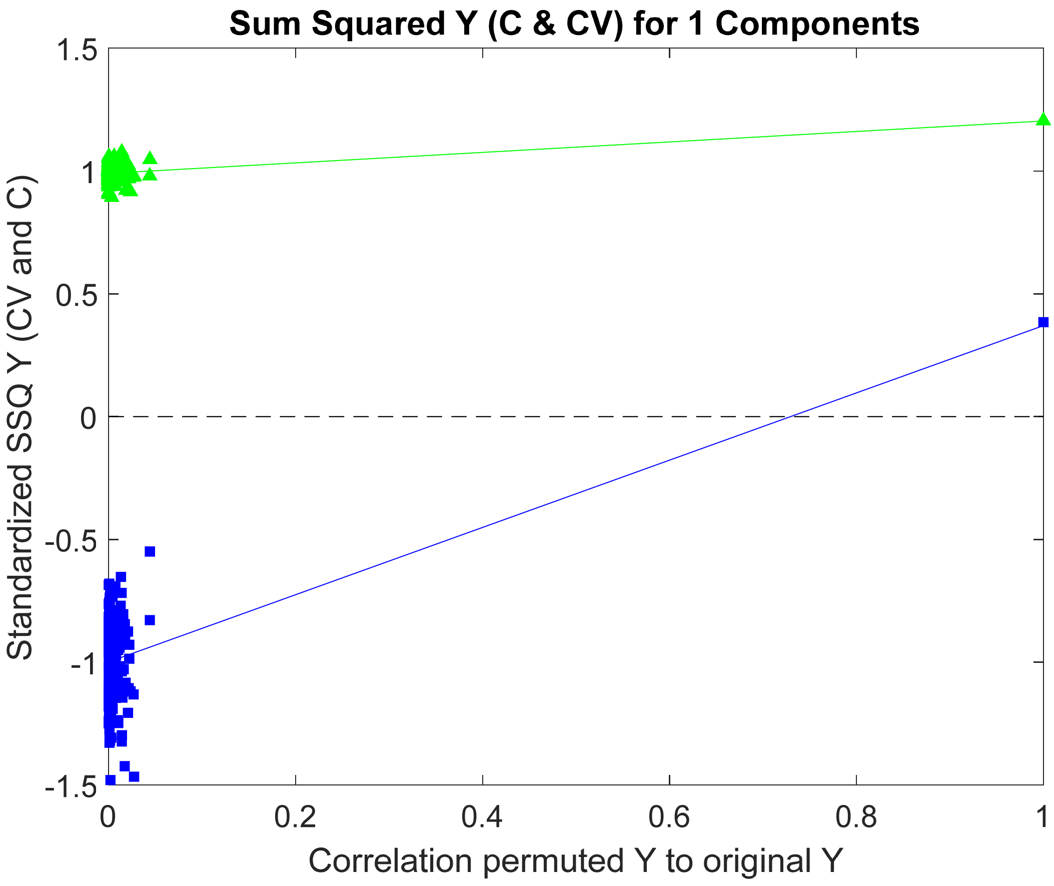

Figure S3h. Permutation test (n=200) of (a) XGB-R model where the R^2 prediction = 0.74

Permutation Results

Probability of Model Insignificance vs. Permuted Samples

Y-column: 1

	Wilcoxon	Sign Test	Rand t-test
Self-Prediction:	0.386	0.389	0.626
Cross-Validated:	0.000	0.000	0.005

Values less than 0.05 indicate the model is significant at the 95% confidence level.

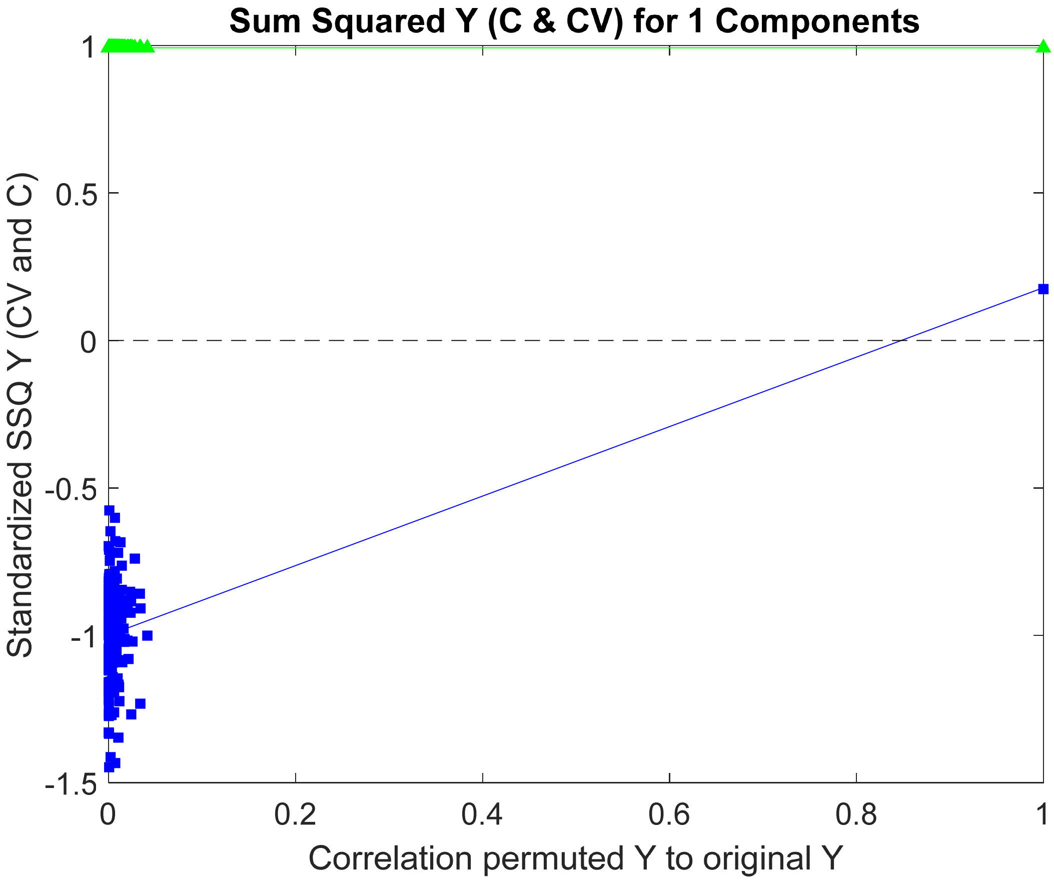

Figure S3i. Permutation test (n=200) of (a) XGB-R model where the R^2 prediction = 0.59

Permutation Results

Probability of Model Insignificance vs. Permuted Samples

Y-column: 1

	Wilcoxon	Sign Test	Rand t-test
Self-Prediction:	0.001	0.005	0.010
Cross-Validated:	0.000	0.000	0.005

Values less than 0.05 indicate the model is significant at the 95% confidence level.

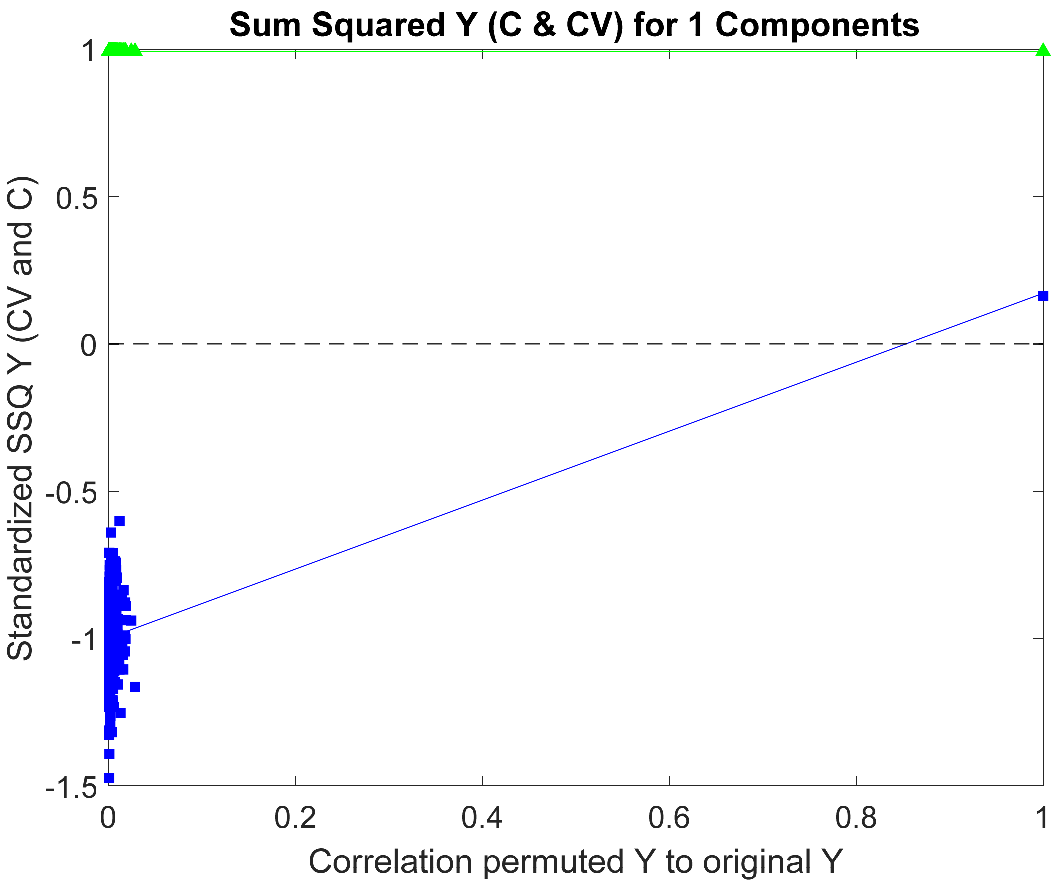

Figure S3j. Permutation test (n=200) of (a) XGB-R model where the R^2 prediction = 0.74
