# Supplementary material for: Rapid In Situ Near-Infrared Assessment of Tetrahydrocannabinolic Acid in Cannabis Inflorescences before Harvest Using Machine Learning
Source: Sensors (Basel). 2024 Aug 6;24(16):5081. doi: 10.3390/s24165081 (PMC11360504; doi:10.3390/s24165081)
Supplement: Supplementary file 1 [file sensors-24-05081-s001.zip › Table S1.pdf]

Table S1. The liquid chromatography mass spectra data of CBDA and THCA concentrations, ratios and chemovar classification within the cannabis dataset (n=264).

List ID	CBDA (mg/g)	THCA (mg/g)	CBDA:THCA	Classification
1	0.42	181.48	0.002	High THCA
2	0.39	156.22	0.002	High THCA
3	0.42	184.57	0.002	High THCA
4	0.43	182.41	0.002	High THCA
5	0.47	200.88	0.002	High THCA
6	0.45	186.84	0.002	High THCA
7	0.39	163.95	0.002	High THCA
8	0.44	184.1	0.002	High THCA
9	0.42	165.34	0.003	High THCA
10	0.44	189.11	0.002	High THCA
11	0.39	159.58	0.002	High THCA
12	0.43	173.69	0.002	High THCA
13	0.46	203.21	0.002	High THCA
14	0.46	200.23	0.002	High THCA
15	0.48	190.79	0.003	High THCA
16	0.42	181.59	0.002	High THCA
17	0.45	189.99	0.002	High THCA
18	0.43	174.3	0.002	High THCA
19	0.38	149.41	0.003	High THCA
20	0.42	179.12	0.002	High THCA
21	0.4	159.85	0.003	High THCA
22	0.43	179.9	0.002	High THCA
23	0.31	141.03	0.002	High THCA
24	0.36	164.63	0.002	High THCA
25	0.31	131.9	0.002	High THCA
26	0.4	171.61	0.002	High THCA
27	0.43	192.33	0.002	High THCA
28	0.47	188.73	0.002	High THCA
29	0.43	173.82	0.002	High THCA
30	0.42	159.71	0.003	High THCA
31	0.43	164.39	0.003	High THCA
32	0.47	191.46	0.002	High THCA
33	0.43	184.7	0.002	High THCA
34	0.46	199.87	0.002	High THCA
35	0.45	190.99	0.002	High THCA
36	0.41	182.45	0.002	High THCA
37	0.48	190.95	0.003	High THCA
38	0.42	171.72	0.002	High THCA
39	0.48	184.55	0.003	High THCA
40	0.49	196.17	0.003	High THCA
41	0.46	191.13	0.002	High THCA
42	0.48	195.44	0.002	High THCA

43	0.47	180.87	0.003	High THCA
44	0.39	140.25	0.003	High THCA
45	0.42	162.69	0.003	High THCA
46	0.48	190.84	0.003	High THCA
47	0.45	173.98	0.003	High THCA
48	0.33	123.69	0.003	High THCA
49	0.38	156.1	0.002	High THCA
50	0.37	148.88	0.002	High THCA
51	0.46	195.27	0.002	High THCA
52	0.39	146.33	0.003	High THCA
53	0.41	160.02	0.003	High THCA
54	0.37	148.18	0.002	High THCA
55	0.29	103.71	0.003	High THCA
56	0.31	108.25	0.003	High THCA
57	0.28	103.1	0.003	High THCA
58	0.34	132.87	0.003	High THCA
59	0.35	131.17	0.003	High THCA
60	0.39	159.69	0.002	High THCA
61	0.41	170.28	0.002	High THCA
62	0.38	149.16	0.003	High THCA
63	0.44	182.83	0.002	High THCA
64	0.44	171.18	0.003	High THCA
65	0.4	153.29	0.003	High THCA
66	0.41	160.12	0.003	High THCA
67	0.45	184.58	0.002	High THCA
68	0.42	169.74	0.003	High THCA
69	0.37	158.56	0.002	High THCA
70	0.41	158.15	0.003	High THCA
71	0.42	173.39	0.002	High THCA
72	0.29	124.1	0.002	High THCA
73	0.3	126.83	0.002	High THCA
74	0.46	183.65	0.002	High THCA
75	0.47	183.58	0.003	High THCA
76	0.45	184.14	0.002	High THCA
77	0.4	162.87	0.002	High THCA
78	0.42	173.45	0.002	High THCA
79	0.43	170.34	0.003	High THCA
80	0.43	175.5	0.002	High THCA
81	0.43	174.68	0.002	High THCA
82	0.48	191.4	0.002	High THCA
83	0.4	148.89	0.003	High THCA
84	0.39	144.75	0.003	High THCA
85	0.45	168.96	0.003	High THCA
86	0.34	104.35	0.003	High THCA
87	0.36	123.73	0.003	High THCA
88	0.29	109.59	0.003	High THCA

89	0.49	182.84	0.003	High THCA
90	0.49	181.38	0.003	High THCA
91	0.44	171.5	0.003	High THCA
92	0.37	130.16	0.003	High THCA
93	0.35	124.37	0.003	High THCA
94	0.34	126.77	0.003	High THCA
95	0.35	130.78	0.003	High THCA
96	0.26	88.33	0.003	High THCA
97	0.26	86.72	0.003	High THCA
98	0.32	110.36	0.003	High THCA
99	0.34	120.3	0.003	High THCA
100	0.38	126.28	0.003	High THCA
101	0.4	140.62	0.003	High THCA
102	0.31	106.69	0.003	High THCA
103	0.36	131.8	0.003	High THCA
104	0.36	135.91	0.003	High THCA
105	0.3	107.69	0.003	High THCA
106	0.29	108.89	0.003	High THCA
107	0.27	92.21	0.003	High THCA
108	0.53	175.69	0.003	High THCA
109	0.46	165.4	0.003	High THCA
110	0.46	179.61	0.003	High THCA
111	0.38	113.19	0.003	High THCA
112	0.38	127.79	0.003	High THCA
113	0.45	133.8	0.003	High THCA
114	0.44	158.16	0.003	High THCA
115	0.37	138.1	0.003	High THCA
116	0.38	148.31	0.003	High THCA
117	0.42	129.37	0.003	High THCA
118	0.34	122.15	0.003	High THCA
119	0.42	135.54	0.003	High THCA
120	0.33	109.45	0.003	High THCA
121	0.39	117.86	0.003	High THCA
122	0.37	129.04	0.003	High THCA
123	0.3	105.78	0.003	High THCA
124	0.35	119.01	0.003	High THCA
125	0.36	128.48	0.003	High THCA
126	0.37	148	0.003	High THCA
127	0.44	161.61	0.003	High THCA
128	0.46	165.73	0.003	High THCA
129	0.42	133.61	0.003	High THCA
130	0.33	114.85	0.003	High THCA
131	0.42	129.79	0.003	High THCA
132	0.39	148.07	0.003	High THCA
133	0.46	172.48	0.003	High THCA
134	0.36	143.48	0.002	High THCA

135	0.34	124.27	0.003	High THCA
136	0.35	130	0.003	High THCA
137	0.39	116.68	0.003	High THCA
138	0.38	104.93	0.004	High THCA
139	0.34	107.52	0.003	High THCA
140	0.32	106.35	0.003	High THCA
141	0.38	132.37	0.003	High THCA
142	0.35	121.67	0.003	High THCA
143	0.36	126.5	0.003	High THCA
144	0.35	135.81	0.003	High THCA
145	0.38	137.85	0.003	High THCA
146	0.36	127.35	0.003	High THCA
147	0.3	102.04	0.003	High THCA
148	0.3	99.51	0.003	High THCA
149	0.34	110.78	0.003	High THCA
150	0.31	88.96	0.003	High THCA
151	0.27	73.23	0.004	High THCA
152	0.3	76.72	0.004	High THCA
153	0.33	114.22	0.003	High THCA
154	0.33	123.73	0.003	High THCA
155	0.36	116.54	0.003	High THCA
156	0.34	116.37	0.003	High THCA
157	0.33	120.39	0.003	High THCA
158	0.34	118.71	0.003	High THCA
159	0.34	131.74	0.003	High THCA
160	0.31	107.24	0.003	High THCA
161	0.34	123.09	0.003	High THCA
162	0.21	37.29	0.006	High THCA
163	0.19	37.51	0.005	High THCA
164	0.49	187.81	0.003	High THCA
165	0.47	171.69	0.003	High THCA
166	0.4	146.21	0.003	High THCA
167	0.51	193.55	0.003	High THCA
168	0.57	204.82	0.003	High THCA
169	0.53	191.06	0.003	High THCA
170	0.35	159.33	0.002	High THCA
171	0.35	168.6	0.002	High THCA
172	0.32	137.34	0.002	High THCA
173	0.44	211.3	0.002	High THCA
174	0.32	145.32	0.002	High THCA
175	0.4	197.69	0.002	High THCA
176	0.36	166.82	0.002	High THCA
177	0.38	187.46	0.002	High THCA
178	0.38	176.61	0.002	High THCA
179	0.45	217.96	0.002	High THCA
180	0.47	211.15	0.002	High THCA

181	0.36	176.79	0.002	High THCA
182	0.34	177.21	0.002	High THCA
183	0.3	131.74	0.002	High THCA
184	0.33	177.22	0.002	High THCA
185	0.43	180.79	0.002	High THCA
186	0.39	201.59	0.002	High THCA
187	0.35	163.87	0.002	High THCA
188	119.18	87.25	1.366	Even Ratio
189	125.5	93.95	1.336	Even Ratio
190	116.17	85.29	1.362	Even Ratio
191	0.39	194.91	0.002	High THCA
192	118.76	98.29	1.208	Even Ratio
193	115.89	72.25	1.604	Even Ratio
194	132.09	80.8	1.635	Even Ratio
195	0.33	175.5	0.002	High THCA
196	111.12	69.19	1.606	Even Ratio
197	0.43	214.7	0.002	High THCA
198	0.32	173.24	0.002	High THCA
199	0.36	199.14	0.002	High THCA
200	0.41	208.1	0.002	High THCA
201	0.41	219.72	0.002	High THCA
202	0.47	227.53	0.002	High THCA
203	0.41	208.58	0.002	High THCA
204	111.7	84.92	1.315	Even Ratio
205	112.92	86.16	1.31	Even Ratio
206	128.32	96.59	1.329	Even Ratio
207	124.6	77.51	1.607	Even Ratio
208	135.62	84.03	1.614	Even Ratio
209	131	80.53	1.627	Even Ratio
210	0.48	229.87	0.002	High THCA
211	0.37	200.25	0.002	High THCA
212	0.41	197.43	0.002	High THCA
213	111.52	91.3	1.221	Even Ratio
214	139.22	108.82	1.279	Even Ratio
215	122.75	99.78	1.23	Even Ratio
216	109.26	74.1	1.475	Even Ratio
217	110.14	75.56	1.458	Even Ratio
218	105.51	72.89	1.447	Even Ratio
219	131.07	76.07	1.723	Even Ratio
220	112.87	68.53	1.647	Even Ratio
221	140.19	83.3	1.683	Even Ratio
222	75.17	53.68	1.4	Even Ratio
223	109.36	74.65	1.465	Even Ratio
224	77.85	59.16	1.316	Even Ratio
225	62.45	47.91	1.303	Even Ratio
226	68.58	53.2	1.289	Even Ratio

227	91.71	59.11	1.551	Even Ratio
228	98.2	61.31	1.602	Even Ratio
229	78.93	51.23	1.541	Even Ratio
230	0.38	189.27	0.002	High THCA
231	0.35	171.29	0.002	High THCA
232	0.35	188.32	0.002	High THCA
233	93.07	68.28	1.363	Even Ratio
234	86.45	61.19	1.413	Even Ratio
235	88.99	64.83	1.373	Even Ratio
236	0.32	169.5	0.002	High THCA
237	0.31	154.51	0.002	High THCA
238	0.37	182.66	0.002	High THCA
239	0.39	208.29	0.002	High THCA
240	0.43	211.66	0.002	High THCA
241	0.44	211.78	0.002	High THCA
242	0.43	215.8	0.002	High THCA
243	0.36	194.91	0.002	High THCA
244	0.35	182.82	0.002	High THCA
245	0.34	178.85	0.002	High THCA
246	0.28	164.25	0.002	High THCA
247	0.51	210.42	0.002	High THCA
248	0.31	143.65	0.002	High THCA
249	0.36	158.95	0.002	High THCA
250	0.33	154.56	0.002	High THCA
251	0.4	177.07	0.002	High THCA
252	0.37	163.54	0.002	High THCA
253	0.39	184.13	0.002	High THCA
254	0.26	124.72	0.002	High THCA
255	0.31	142.58	0.002	High THCA
256	0.46	197.84	0.002	High THCA
257	0.36	181.45	0.002	High THCA
258	0.45	214.1	0.002	High THCA
259	0.3	159.5	0.002	High THCA
260	0.18	95.62	0.002	High THCA
261	0.41	179.64	0.002	High THCA
262	0.47	199.07	0.002	High THCA
263	0.51	226.6	0.002	High THCA
264	0.53	221.59	0.002	High THCA
