# Supplementary material for: Rapid In Situ Near-Infrared Assessment of Tetrahydrocannabinolic Acid in Cannabis Inflorescences before Harvest Using Machine Learning
Source: Sensors (Basel). 2024 Aug 6;24(16):5081. doi: 10.3390/s24165081 (PMC11360504; doi:10.3390/s24165081)
Supplement: Supplementary file 1 [file sensors-24-05081-s001.zip › Table S2.pdf]

Table S2. Standard error laboratory versus the standard error prediction for THCA (n = 264).

	**SEL (mg/g)**	**SEP (mg/g)**	**SEP/SEL**	**R^2**	**Mean Cannabinoid Concentration from LCMS data (mg/g)**
**THCA**	3.03	21.49	7.08	0.78	147.63
