# Supplementary material for: Rapid In Situ Near-Infrared Assessment of Tetrahydrocannabinolic Acid in Cannabis Inflorescences before Harvest Using Machine Learning
Source: Sensors (Basel). 2024 Aug 6;24(16):5081. doi: 10.3390/s24165081 (PMC11360504; doi:10.3390/s24165081)
Supplement: Supplementary file 1 [file sensors-24-05081-s001.zip › Table S3.pdf]

Table S3. Error of prediction values for the validation dataset used to test the PLS (R^2=0.78) prediction model

Sample Validation ID	Conc. (mg/g)	Error (mg/g)	%Error
1	181.48	-14.0	-8%
2	189.11	-0.9	0%
3	159.58	23.0	14%
4	200.23	-17.0	-9%
5	149.41	19.3	13%
6	188.73	-24.2	-13%
7	184.7	-10.5	-6%
8	184.55	-10.8	-6%
9	103.71	17.8	17%
10	184.58	-25.3	-14%
11	173.39	-6.3	-4%
12	183.65	0.9	0%
13	184.14	-47.4	-26%
14	170.34	8.3	5%
15	174.68	-3.5	-2%
16	191.4	-16.3	-9%
17	104.35	29.7	28%
18	181.38	-29.6	-16%
19	88.33	30.9	35%
20	86.72	17.7	20%
21	110.36	33.6	30%
22	165.4	-7.5	-5%
23	179.61	-34.5	-19%
24	135.54	3.4	3%
25	129.04	9.0	7%
26	148	8.5	6%
27	148.07	25.2	17%
28	104.93	-8.1	-8%
29	107.52	18.7	17%
30	106.35	26.4	25%
31	88.96	61.8	69%
32	116.54	31.3	27%
33	120.39	13.2	11%
34	118.71	19.4	16%
35	171.69	-19.0	-11%
36	146.21	-16.4	-11%
37	193.55	-42.3	-22%
38	191.06	-28.0	-15%
39	159.33	-15.5	-10%
40	211.3	-31.9	-15%
41	177.21	6.8	4%
42	163.87	42.0	26%

43	87.25	3.9	5%
44	93.95	1.2	1%
45	194.91	-26.7	-14%
46	72.25	-0.5	-1%
47	175.5	-12.2	-7%
48	214.7	-17.9	-8%
49	86.16	-6.5	-8%
50	84.03	19.7	23%
51	80.53	15.0	19%
52	200.25	-26.2	-13%
53	197.43	-16.8	-9%
54	76.07	-8.9	-12%
55	83.3	-19.9	-24%
56	74.65	15.5	21%
57	61.31	-8.1	-13%
58	189.27	-7.2	-4%
59	68.28	5.9	9%
60	169.5	5.0	3%
61	154.51	11.7	8%
62	182.66	-19.4	-11%
63	194.91	-1.9	-1%
64	164.25	-0.2	0%
65	178.85	4.1	2%
66	214.1	-44.1	-21%
