# Supplementary material for: Rapid In Situ Near-Infrared Assessment of Tetrahydrocannabinolic Acid in Cannabis Inflorescences before Harvest Using Machine Learning
Source: Sensors (Basel). 2024 Aug 6;24(16):5081. doi: 10.3390/s24165081 (PMC11360504; doi:10.3390/s24165081)
Supplement: Supplementary file 1 [file sensors-24-05081-s001.zip › Table S4.pdf]

Table S4. The partial least squares regression (PLS-R) models for the determination of THCA concentration where the water peak region (1400 – 1450 nm) from the NIR spectra has been removed.

Model	Region (nm) [1]	Scatter Correction [2]	Derivative [2a]	N [3]	RMSEC [4]	R^2_{Cal} [5]	RMSECV [6]	R^2_{CV} [7]	RMSEP [8]	Pred Bias [9]	R^2_{Pred} [10]
PLS-R	950 – 1400, -<br>1450 - 1650	DT, SNV and MC	2, 2, 5	264	24.06	0.70	25.32	0.67	26.38	-0.38	0.58

[1]nm: wavelength in nanometres.

[2]Preprocessing parameters. DT: detrend; SNV: standard normal variate; MC: mean centering;

[2a]Derivative pre-treatment: the first digit is the polynomial order, the second digit is the derivative order and the third digit is the data point gap which the derivative is calculated.

[3]N: number of unique samples.

[4]RMSEC: root mean standard error of calibration.

[5]R^2_{Cal}: coefficient of determination of calibration.

[6]RMSECV: root mean standard error of cross validation.

[7]R^2_{CV}: coefficient of determination of cross validation.

[8]RMSEP: root mean standard error of prediction.

[9]Pred Bias: calculated prediction bias.

[10]R^2_{Pred}: coefficient of regression of measured data vs predicted data; Permutation testing (n = 50) using Wilcoxon test returned p-value < 0.05.
